# Insights into *Acinetobacter baumannii* protective immunity

**DOI:** 10.3389/fimmu.2022.1070424

**Published:** 2022-11-18

**Authors:** Sean Jeffreys, James P. Chambers, Jieh-Juen Yu, Chiung-Yu Hung, Thomas Forsthuber, Bernard P. Arulanandam

**Affiliations:** ^1^ Department of Molecular Microbiology and Immunology, University of Texas at San Antonio, San Antonio, TX, United States; ^2^ Department of Immunology, Tufts University School of Medicine, Boston, MA, United States

**Keywords:** Acinetobacter baumannii, adaptive immunity, antibody-mediated protection, cell-mediated protection, immunotherapeutic, vaccine

## Abstract

*Acinetobacter baumannii* is a nosocomic opportunistic Gram-negative bacteria known for its extensive drug-resistant phenotype. *A. baumannii* hospital-acquired infections are major contributors to increased costs and mortality observed during the COVID-19 pandemic. With few effective antimicrobials available for treatment of this pathogen, immune-based therapy becomes an attractive strategy to combat multi-drug resistant *Acinetobacter* infection. Immunotherapeutics is a field of growing interest with advances in vaccines and monoclonal antibodies providing insight into the protective immune response required to successfully combat this pathogen. This review focuses on current knowledge describing the adaptive immune response to *A. baumannii*, the importance of antibody-mediated protection, developments in cell-mediated protection, and their respective therapeutic application going forward. With *A. baumannii*’s increasing resistance to most current antimicrobials, elucidating an effective host adaptive immune response is paramount in the guidance of future immunotherapeutic development.

## Introduction

The incidence of multi-drug-resistant (MDR) bacteria strains increases yearly; thus, alternative therapies are required to compensate for increasingly ineffective antimicrobials that are currently available. An increasingly clinically relevant MDR pathogen is *Acinetobacter baumannii*, an opportunistic nosocomial Gram-negative bacterium, and member of the ESKAPE group of pathogens which include *Enterococcus faecium, Staphylococcus aureus, Klebsiella pneumoniae, Pseudomonas aeruginosa*, and *Enterobacter* species (1). The ESKAPE designation refers to an extensive drug-resistant phenotype, and ability to overcome most current antimicrobial therapies. The prevalence of cases of *A. baumannii* infection is increasing worldwide and is a leading cause of morbidity and mortality (2, 3). This has prompted the World Health Organization (WHO) to assign carbapenem-resistant *A. baumannii* the highest critical priority ranking emphasizing the urgent need to identify new and effective drug therapies to combat this pathogen (4).

The opportunistic nature of *A. baumannii* primarily affects at-risk hospitalized patients with stays of longer-duration, especially immunocompromised individuals (5). Additionally, it is increasingly the source of infection in combat-wounded military personnel (6, 7). *A. baumannii* infection commonly presents as ventilator-associated pneumonia and catheter-related bloodstream/urinary infection, in addition to that of wounds (8–10). Due to their MDR nature, empiric treatment is generally ineffective and results in poor clinical outcomes (11). If untreated, rampant bacterial growth can ensue and give rise to TLR4-mediated septic shock and death (12, 13).

The incidence of MDR *A. baumannii* co-infection has increased in intensive care units (ICU) due to prolonged hospitalization of COVID-19 patients (14). One retrospective study of *A. baumannii* co-infection in COVID-19 hospitalized patients in Wuhan, China found that nearly half of the individuals who developed secondary bacterial infection died, with *A. baumannii* accounting for 36% of these infections (15). In another study, nearly 12% of ICU patients admitted with severe COVID-19 pneumonia developed secondary bacterial infections with *K. pneumoniae* or *A. baumannii*. Both pathogens exhibited extensive MDR resulting in more than double the mortality rate (from 38 to 83%) typically observed in COVID-19 patients (16). The incidence of *A. baumannii* infection in the past decade is associated with increased ICU admission, and longer hospital stays (17, 18). The nosocomic nature of this pathogen arises from its resistance to desiccation, and its ability to thrive in environments under selective pressures (19).

Investigation of *A. baumannii* pathogenic mechanisms and development of effective therapeutics against this pathogen has been a long-standing clinical and laboratory challenge. Initially, clinical isolates exhibited a hypovirulent phenotype necessitating the use of immunosuppressed mice as hosts or mixing of bacteria with porcine mucin to inhibit initial host clearance to study its pathophysiological mechanisms (20). However, recent MDR clinical isolates exhibit a hypervirulent phenotype, which rendered immunocompetent wild-type mice more readily susceptible to bacterial challenge (21–23). Thus, immunocompetent hosts now afford useful models to assess the efficacy of novel therapeutics with these more virulent *A. baumannii* isolates.

In order to develop novel and effective therapies to combat this pathogen it is first necessary to elucidate the underlying immune mechanisms by which *A. baumannii* evades the host’s adaptive and innate immune system. In this review we examine the current understanding of antibody-mediated and cell-mediated immune responses and key elements of protective immunity derived from recent therapeutic studies.

## Antibody-mediated protection to *A. baumannii*

The success of passive immunization and use of monoclonal antibodies underscores the essential role of antibody-mediated immunity in protection against *A. baumannii* (24–33). Consistent with this notion, *A. baumannii* vaccinated B cell knockout mice were not protected in a murine-pneumonia model (34). Compared to vaccinated wild-type animals, these mice developed higher bacterial loads and subsequent extrapulmonary bacterial dissemination and bacteremia.

Moreover, some studies show correlation of survival with specific antibody titers (26, 35). Dose variation in subunit vaccines has shown larger doses of recombinant *A. baumannii* protein elicit higher antibody titers and higher protective efficacy (30). Interestingly, some studies observed higher protection in passive than in active immunization models (28, 36, 37). Across most studies, passive immunization has been shown to be an effective therapeutic route with only one study using an intranasal challenge model showing no improvement in survival albeit a delayed time to death (34). Thus, a focus of this review is that of the protective role of antibody-mediated immunity to *A. baumannii*.

### Antibody isotype

Consistent with the theme that antibody-mediated protection is the primary driver of *A. baumannii* vaccine protection, most studies have shown that whole-cell and outer membrane vesicle (OMV) vaccines are capable of generating high *A. baumannii* specific IgG (including both IgG1 and IgG2) antibody titers through various immunization routes (28, 32, 34, 35). Additionally, intranasal immunization produces significant serum levels of sIgA exhibiting higher protective efficacy than that of intramuscular immunization in an intratracheal challenge model (35). In an intramuscular vaccination study examining OMV protection, OMV-specific IgA was observed significantly increased in the serum and bronchoalveolar lavage fluids following intranasal challenge (28). These two studies demonstrate the importance of *A. baumannii-*specific sIgA production for mucosal immunity in respiratory infections. Interestingly, a respiratory infection model utilizing an FcRγ knockout mouse strain afforded 100% survival in vaccinated groups, suggesting that IgG is not essential for protection in *A. baumannii* lung infection models, and that IgA could be sufficient for mucosal immunity (34). To the contrary, sIgA has been shown to enhance *A. baumannii* gastrointestinal tract colonization leaving its role in mucosal immunity to be further assessed (38), especially with regard to the numerous clinical manifestations of ventilator-associated pneumonia.

### Antibody-mediated complement activation

An essential innate immune component is the complement system which works synergistically with the adaptive immune response to enhance pathogen clearance. Complement activation can occur *via* three different pathways: the classical, lectin, and alternative complement pathways. The lectin and alternative pathways entail direct interaction of complement with the pathogen’s cell surface in contrast to the classical pathway which first requires interaction with the Fc region of bound antibody to the bacterial cell surface. The classical pathway’s antigen-antibody complex connects the innate effector function of complement with that of the antigenic specificity of the humoral immune response. Microbe-bound antibodies synergistically enhance the complement cascade, and promote downstream innate effector functions, i.e., production of proinflammatory molecules such as C3a, C4a, and C5a, the formation of the membrane attack complex, and opsonization of microbes with bound C3b. Thus, pathogen-specific antibodies that target bacteria are critical activators of the complement system facilitating pathogen clearance (39).

The antigen specific nature of the classical pathway has led to extensive investigations of its role in antibody-mediated protection. *A. baumannii*’s outer membrane plays a critical role in its MDR nature and is a potential target for complement-mediated membrane attack complex formation (40). However, the role of complement in this mechanism of protection is difficult to study due to *A. baumannii* exhibiting variability in susceptibility to complement activation across its many strains (41, 42). A common mechanism of complement resistance is that of the pathogen’s recruitment of the host’s complement regulators to its surface and inhibition of complement activation (43). The most studied *A. baumannii* virulence factor, OmpA, enhances complement resistance by interacting with Factor H to down-regulate complement activation on the pathogen’s surface (44). Interestingly, a recombinant OmpA vaccine did not abrogate or neutralize OmpA’s complement inhibitory function, and immune serum did not result in enhanced complement-mediated killing of *A. baumannii* (26). This contrasts with other studies showing that vaccinated immune sera enhanced direct *A. baumannii* killing activity with intact complement, but this may have been due to the use of complement-sensitive strains (25, 45). This suggests that complement resistant strains are insensitive to direct bactericidal killing *via* formation of the membrane attack complex even in the presence of *A. baumannii* specific antibodies.

**Figure 1:**
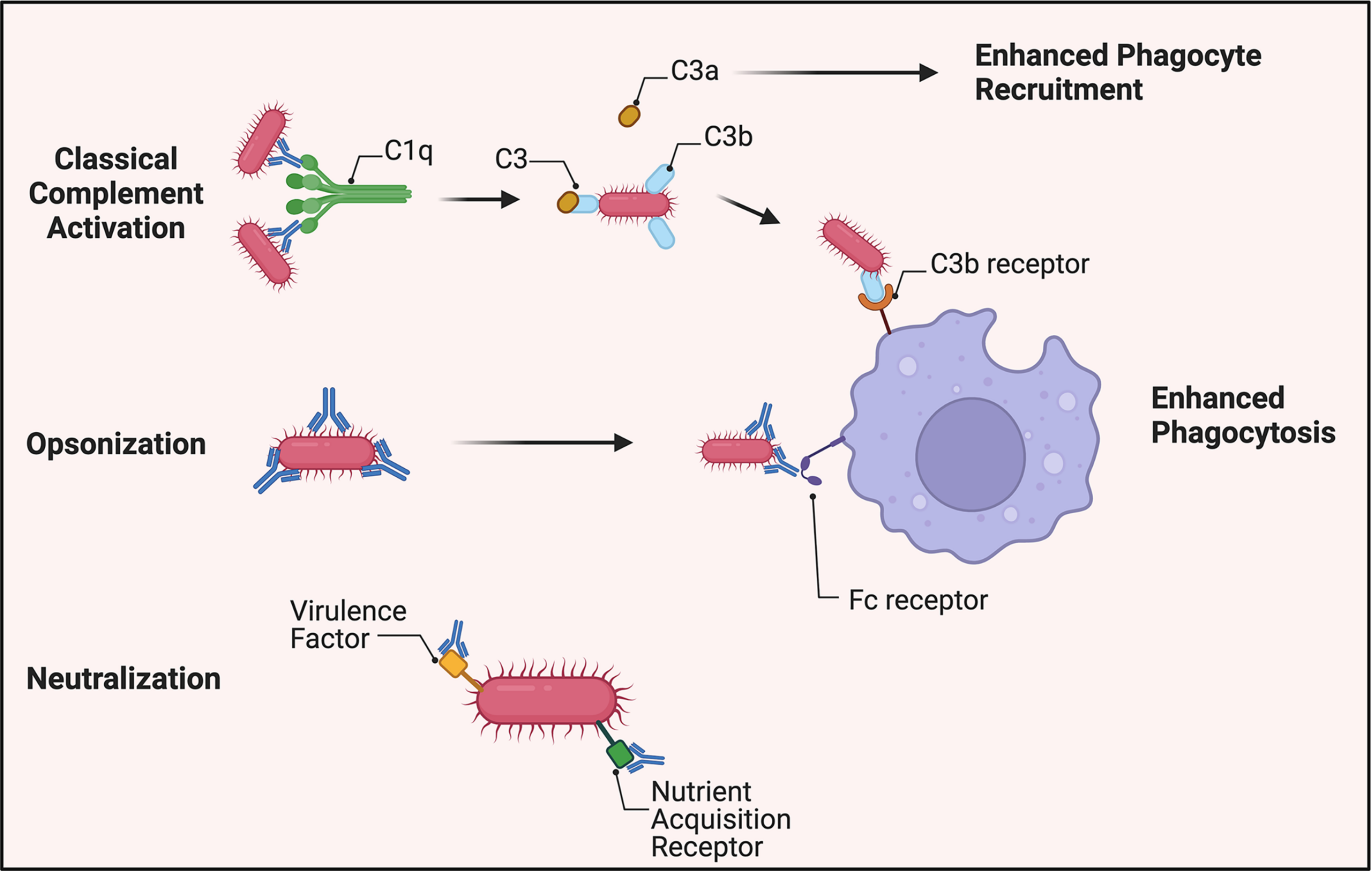
Overview of antibody-mediated protection against *A. baumannii.* Virulent strains appear to be resistant to direct bactericidal activity *via* membrane attack complex formation. *A. baumannii*-specific antibodies, however, still mark the pathogen for C3b deposition on its surface by activation of the classical complement pathway. C3a is released to increase phagocyte recruitment to the site of infection. In the absence of complement, antibodies continue to identify the bacterium for increased phagocytosis through antibody-Fc receptor interactions. Additionally, antibodies generated toward virulence factors required for pathogenesis, such as siderophores, can attenuate *A. baumannii*’s virulence.

Of note, however, studies show a reduction in opsonophagocytic enhancement *via* vaccinated sera when complement is removed through heat inactivation (28–30). This suggests that complement may play a role in *A. baumannii* control *via* opsonization and enhanced phagocytosis through C3b deposition on the pathogen’s surface as well as release of C3a to promote recruitment of innate effector cells (**Figure 1**). A recent study of an *A. baumannii* specific monoclonal antibody showed that the antibody’s efficacy was linked to complement activation, and administration of the mAb led to increased serum levels of C3a consistent with synergistic activation of the classical pathway. This conclusion was further supported by reduced efficacy of the mAb in C3 knockout mice, indicating complement C3 acts in tandem with the humoral immune response to clear *A. baumannii* (46). Collectively, these data suggest that complement is not essential for antibody-mediated immunity but improves the host’s capability to clear *A. baumannii via* opsonization and phagocytosis.

### Antibody opsonization

Opsonization occurs *via* binding of specific antibodies to bacteria thus enhancing their clearance by phagocytes through antibody-Fc receptor(FcR)-mediated phagocytosis (47).

Early studies demonstrated that incubation of *A. baumannii* with monoclonal antibodies enhanced their opsonophagocytic uptake by human polymorphonuclear cells (48). Many *A. baumannii* vaccine studies show that innate effector cells are critical for bactericidal activity, and in their absence, antiserum alone has no bactericidal effect (29, 30). Additionally, the presence of *A. baumannii-*specific antibodies significantly increased uptake by macrophages (**Figure 1**). A monoclonal antibody-based study underscored the importance of the interaction of the antibody Fc region with FcR. The protective efficacy of the mAb generated against *A. baumannii* capsular polysaccharide decreased from 100 to 20% upon conversion to f(ab)_2_ fragments lacking an Fc region. The inability to facilitate the Fc-FcR interaction resulted in evasion of neutrophil and macrophage-mediated phagocytosis leading to fulminant *A. baumannii* growth, and subsequent lethal sepsis (46). These data are supportive of the essential protective role of antibody-mediated immunity to *A. baumannii*, i.e., the targeting nature of antibodies and enhancement of opsonization of the bacteria by innate cells.

### Neutralization

A much understudied aspect of antibody-mediated immunity to *A. baumannii* is that of neutralization capability. Traditional antibody neutralization targets, *e.g.*, secreted microbial toxins are not well-characterized with regard to *A. baumannii*. However, some *A. baumannii* virulence factors can be neutralized by antibodies (**Figure 1**). For example, antiserum generated to the virulence factor Ata (a surface autotransporter protein) that binds to basal membrane proteins and the extracellular matrix prevented adhesion of *A. baumannii* to collagen type IV (25, 49). In another study, *A. baumannii* iron sequestering proteins, i.e., siderophores were targeted using antiserum generated against iron-regulated outer membrane proteins and was shown to be bactericidal when the bacterium was incubated in iron-deficient media (48, 50). These studies support further the contention that antibody targeting of virulence factors upregulated during pathogenesis is a viable therapeutic strategy.

### Monoclonal antibodies

The protective role of humoral immunity to *A. baumannii* is well-established; therefore, development of monoclonal antibodies (mAb) against this pathogen have been pursued as potential therapeutic agents. In this vein, a mAb generated against the K1 polysaccharide capsule of *A. baumannii* enhanced neutrophil-mediated opsonophagocytosis of K1 positive strains. Post-challenge administration of the mAb was protective and reduced bacterial burdens in a soft tissue infection model in rats (27). However, only 13% of the *A. baumannii* strains tested were seropositive for the K1 capsule which limits broad use of this mAb as a therapeutic reagent. In a subsequent study, mAb generated against the capsule of more recently isolated hypervirulent strains, bound to 39% of 302 total strains tested (33). This mAb protected mice following both blood and lung challenge suggesting that a broadly cross-reactive monoclonal antibody could be a viable therapy against acute MDR *A. baumannii* infections in the clinic.

## Cell-mediated protection to *A. baumannii*

Much of the vaccine development efforts for *A. baumannii* have focused on antibody-mediated immunity, and few studies have investigated the cell-mediated aspects of protection. As a result, most studies have utilized the IgG isotype ratio to determine whether vaccine-induced protection was Th1 or Th2 driven, and few studies have investigated direct T cell responses to *A. baumannii*, *e.g.*, in T cell recall and adoptive transfer.

### Antibodies as surrogate indicators of protection provided by specific T cell subsets

**Figure 2:**
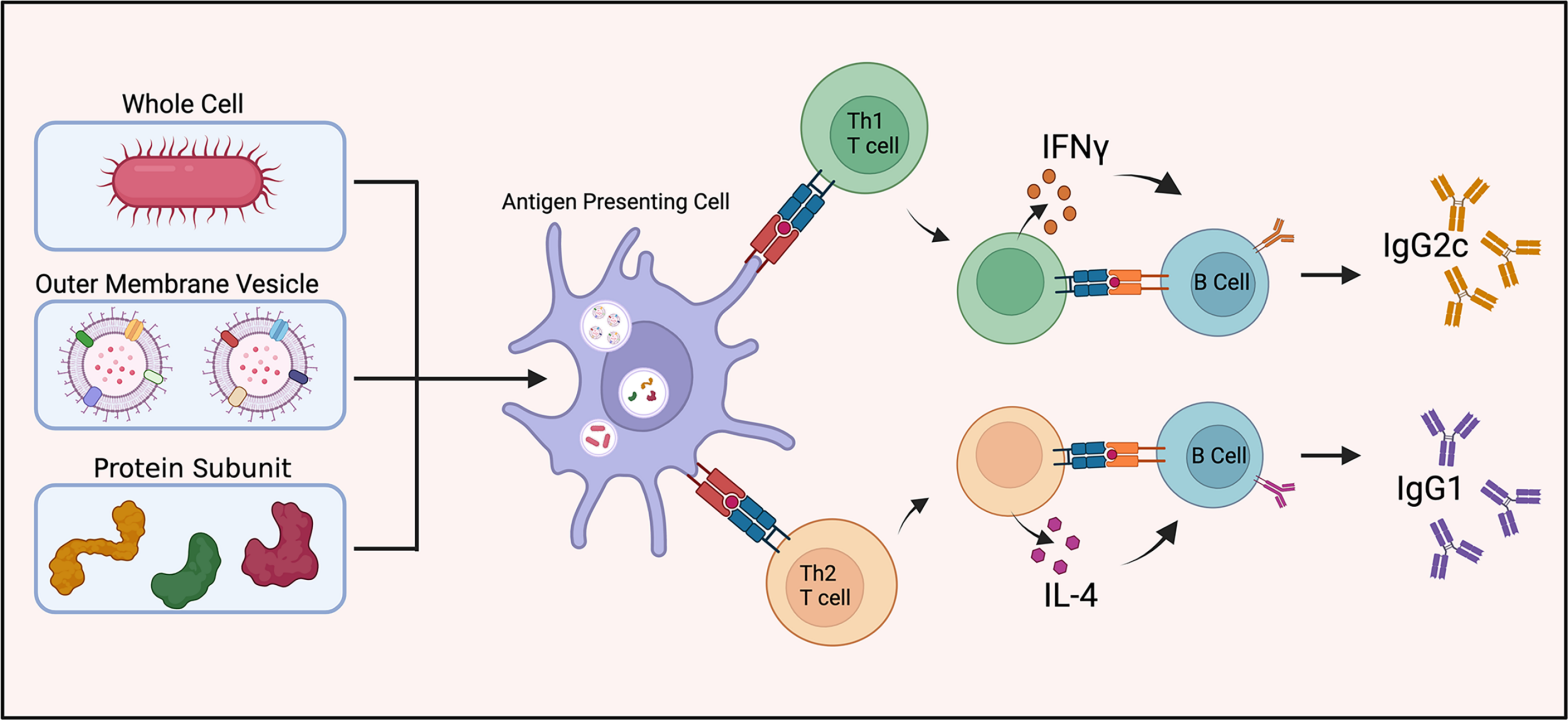
Vaccine induced antibody generation. Schematic illustration of antibody generation from inactivated and live attenuated whole cell, outer membrane vesicle, and protein subunit vaccines towards *A. baumannii*. The proportion of IgG1 to IgG2c antibody production is utilized as a surrogate indicator of Th1 or Th2 mediated immunity.

Antibody isotype production can aid as a surrogate indicator as to the immune response being mediated by specific T cell subsets, *e.g*. Th1 or Th2 immunity. IFN-γ (a Th1 cytokine) and IL-4 (a Th2 cytokine) stimulate switching of immunoglobulin isotype in B cell proliferation and differentiation (**Figure 2**). IFN-γ induces production of IgG2a/IgG2c while IL-4 stimulates IgG1 production (51). The ratio of IgG1 to IgG2a (or IgG2c) functions as an indirect indicator of Th1 or Th2 mediated immune response in mice.

Studies using whole-cell inactivated and/or live attenuated strains of *A. baumannii* showed robust IgG1 and IgG2a (or IgG2c) antibody responses indicating both Th1 and Th2 cell engagement (24, 34, 52). Vaccination with *A. baumannii* OMVs elicits a balanced Th1 and Th2 response based on this ratio (28, 53). One study found OMV vaccines generate a different immune response dependent on the route of vaccination. Intramuscular immunization led to a balanced Th1 and Th2 response while intranasal immunization exhibited uniformly a Th1 (IgG2a) biased response (35). Collectively, these studies provided evidence for a balanced adaptive immune response to a broad range of *A. baumannii* antigens.

### T cell responses

Several studies have directly measured T cell involvement in vaccine induced protection as well as *A. baumannii’*s influence on antigen presenting cells responsible for priming of the T cell response. One study examined T cell responses in splenocytes obtained from mice immunized with a live-attenuated vaccine (D-glutamate auxotrophic Δ*murI1*Δ*murI2*), and measured IFN-γ, IL-4, and IL-17 secretion as indicators of Th1, Th2, or Th17 responses by cytokine ELISPOT assay, respectively (32). Of note, this study reported a significant elevation of IL-4 and IL-17, but not IFN-γ by T cells after stimulation with the vaccine strain supporting that protective live-attenuated vaccine induce a Th2 and Th17 response. Moreover, an OMV vaccine-based study exposed bone marrow-derived dendritic cells (BMDC) to *A. baumannii* OMVs and found an activated phenotype with upregulation of costimulatory molecules and production of proinflammatory cytokines (35). Another OMV-based vaccine study investigated the BMDC response as well as the resulting T cell populations in vaccinated mice (54). Exposure to the OMV increased costimulatory molecules expressed by BMDC and production of IL-4. In agreement, splenocytes of vaccinated mice exhibited an increase in the Th2 subset (CD4^+^/IL-4^+^) with no change in IFN-γ and IL-17. Of note, this study compared the OMVs produced from a standard strain (ATCC19606) with the clinical isolate (JU0126). The Th2 polarization was observed with the clinical isolate but not with ATCC19606, suggesting that variance in cell-mediated response depends on the strain of *A. baumannii* used, and the variability of antigens during exposure.

The variance in immunogenicity of *A. baumannii* strains is central to the difficulty in establishing an optimized conserved protective immune response thus far. However, recent protein subunit vaccines are beginning to elucidate the responses to specific virulence factors. OmpA, a well-studied virulence factor and focus of many vaccination studies has demonstrated a Th1 polarizing ability *in vitro*. OmpA-exposed dendritic cells stimulated CD4^+^ T cell secretion of IFN-γ (55). Interestingly, vaccination with recombinant OmpA exhibited a dose-dependent effect, i.e., a 3 µg dose induced a balanced Th1/Th2 response, while a 100 µg dose gave a biased Th2 response following splenocyte restimulation (56). Thus additional complexity is added to the question of optimal cell mediated immunity with the addition of vaccine adjuvants and dose variance among protein subunit vaccines.

Whether Th1, Th2, or Th17 responses or balanced T cell cytokine responses are preferential for *A. baumannii* protection remains unanswered and requires further investigation. Considering the central role of humoral immunity for *A. baumannii* protection, a strong Th2 response could be essential to provide B cell help. Thus far, the use of antibodies as surrogate indictors of an indirect Th1 or Th2 response paints a picture favoring a balanced response, while the relatively few studies directly investigating T cell cytokine profiles have displayed preference for more biased responses. Overall, elucidation of the critical role of T helper cells in activation of the humoral response requires additional studies.

## Vaccine-induced protective immunity

With the increasing drug-resistance of *A. baumannii* and few new antibiotic prospects on the horizon, vaccines have been looked to as a potential preventative measure for the most ‘at-risk’ patients, i.e., those developing infection due to prolonged hospitalization. Many vaccine studies utilized live-attenuated *A. baumannii* strains, inactivated whole-cell vaccines, or OMV preparations due to their robust immunogenicity and ability to induce protection (24, 28, 32, 34, 52). With the emergence of transcriptomics and identification of essential *A. baumannii* virulence factors, more emphasis has been placed on the development of recombinant protein subunit vaccines with improved safety profiles (26, 29, 30, 37). Along these lines, next-generation vaccine technology has been applied to engineering DNA vaccines against *A. baumannii* virulence factors that have proven difficult to produce for subunit vaccines (57, 58).

**Figure 3:**
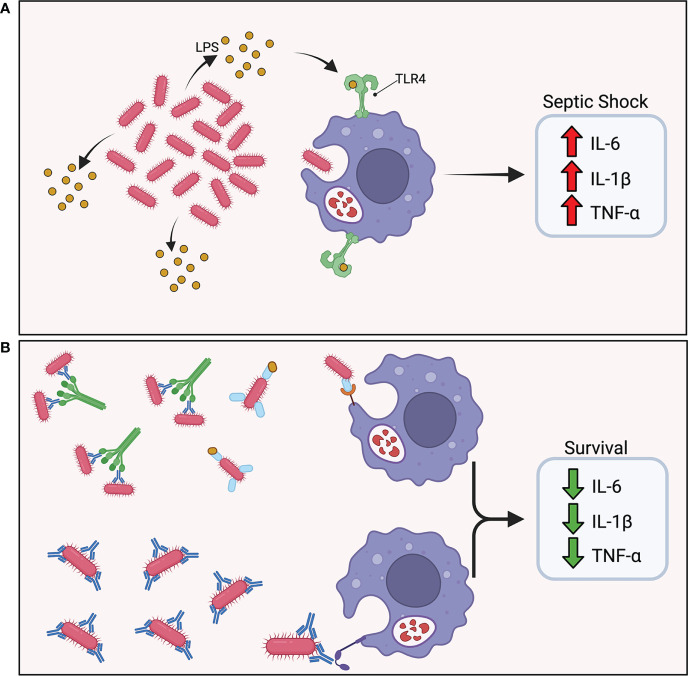
Vaccine induced protective immunity. **(A)** In the absence of protective immunity, hypervirulent *A*. *baumannii* rapidly proliferates in the host leading to excess shedding of lipopolysaccharide (LPS) and rampant TLR4 activation. The abundance of pro-inflammatory cytokine secretion results in a cytokine storm and ultimately septic shock. **(B)** In the presence of protective immunity, antibodies mark the pathogen for phagocytic uptake by innate effector cells. A rapid reduction in bacterial burden maintains a balanced immune response.

Vaccine-induced protective immunity tends to correlate with lower bacterial burdens 12 to 24 hours post-challenge in models using hypervirulent strains of *A. baumannii* that typically lead to acute death within 24 to 48 hours (12, 29, 31, 32, 52, 53). These studies utilize intravenous and intraperitoneal injections of bacterial inoculum to simulate bacteremia and septic shock and have demonstrated vaccine-induced protection through rapid clearance of bacteria (**Figure 3**). Additionally, an *A. baumannii*-associated pneumonia model showed reduced bacterial burden in the lungs as well as limiting bacterial dissemination to extrapulmonary areas in vaccinated mice 24 hours after intranasal challenge (34). The acute nature of these models underscores the central role that preexisting antibody-mediated immune responses play in clearance of *A. baumannii* prior to bacterial dissemination. The abrogation of cytokine storm as a result of rapid bacterial clearance resulted in the drastic reduction in proinflammatory cytokine levels in protected mice, and a significant decrease was noted in IL-1β, TNF-α, and IL-6 in serum 12 and 24 hours post-challenge in protected mice (12, 24, 29, 34, 36, 52, 53). This was observed with both active and passive immunization.

The effect of vaccine-induced protective immunity on reducing *A. baumannii* pathology underscores the importance of early control and rapid clearance of the infection. Specifically, early time points post-challenge displayed pathology similar to mock-vaccinated animals followed by drastic reduction in inflammation 24 hours post-challenge (28, 31). Moreover, vaccinated mice exhibited limited infiltration of neutrophils and macrophages in lung infection models (21, 59), and active and passive immunization studies documented the importance of controlling bacterial burden within 24 hours of challenge.

Interestingly, a recent study utilizing a protective mAb in combination with complement, macrophage, and neutrophil depletion to evaluate the protective mechanisms showed that correlation between survival and bacterial burden does not always apply (46). In this study, double and even triple depleted mice did not survive subsequent challenge, but still exhibited strikingly reduced bacterial burdens compared to placebo mice. Use of TNF-α knockout mice indicated that the cytokine was not required for mAb efficacy. Moreover, IL-10 knockout mice were not protected which is in accordance with previous studies that have demonstrated its importance in *A. baumannii* infection (60, 61). However, macrophage depletion or treatment with an anti-TNFα antibody restored the efficacy of the mAb in IL-10 knockout mice. Efficacy of the mAb appeared to be linked to induction of IL-10 from neutrophils which balanced the macrophage’s TNF-α rather than simply reduction in bacterial burden observed in other therapeutic studies. The authors concluded that the protection conferred by the mAb was a result of inflammatory cytokine modulation suggesting that the delicate equilibrium of the innate and adaptive immune response is required for *A. baumannii* clearance, and that imbalance elicits a cytokine storm detrimental to the host. This interconnection remains to be elucidated as alternative therapeutics for *A. baumannii* infection are developed.

## Conclusion and opinion

*Acinetobacter baumannii* is an increasingly relevant opportunistic pathogen due to development of MDR. The World Health Organization has assigned carbapenem-resistant *A. bau*mannii as the ‘rank one’ critical pathogen requiring new therapeutic identification and development. With few new antibiotics on the horizon, novel therapies like vaccines and monoclonal antibodies must be developed to deal with this urgent microbial threat. Immunotherapeutic development has shed light on the potential mechanisms of adaptive protection against *A. baumannii*. Current research has demonstrated the importance of antibody-mediated protection through passive transfer of serum antibodies, B cell knockouts, and generation of monoclonal antibodies. The efficacy of this protection appears to be primarily a result of antigen-specific coating of the pathogen leading to opsonophagocytosis by neutrophils and macrophages. The role of cell-mediated immunity in this protection remains incompletely understood and will require further study to determine the exact underlying mechanisms. Protective efficacy correlates with lower bacterial burdens in addition to reduced proinflammatory cytokine production. However, recent studies have investigated immunomodulation of these therapeutics in maintaining immune homeostasis and dampening of cytokine storm. A greater understanding of how the adaptive immune system counters *A. baumannii* virulence factors in secondary infection can give much needed insight into the most effective immune response required to clear this pathogen.

The advances and research outlined in this review are expected to lead to novel immunotherapeutics to combat *A. baumannii* infections. An effective therapy would impact real world patient mortality and hospital costs arising from nosocomial infection. With few novel antimicrobials in development, this microbe’s ability to become resistant to current therapies outpaces current antibiotic treatment options. The key to the future development and implementation of vaccines and mAbs to *A. baumannii* may be establishing of a conserved immune response across multiple strains. Current research into *A. baumannii* will benefit from using clinically relevant bacterial strains and experimental models. Despite antibody-mediated protection demonstrating success in acute challenge models, this may only incompletely address the importance of cell-mediated immunity to *A. baumannii*. Moreover, it remains to be seen if the protection displayed in animal challenge models is indicative of protection against natural infection in humans. Studies have demonstrated rapid antibody-mediated neutralization of large doses of bacteria, but achieving these antibody titers early during initial bacterial exposure may not be realistic in a clinical setting.

In the laboratory setting, challenges remain in elucidating the mechanisms underlying the adaptive immune responses to *A. baumannii*, for example due to the use of different bacterial strains between laboratories and variations in experimental models used to investigate therapeutic efficacy. *A. baumannii* strains used to induce protection in certain experimental animal models may not provide protection against infection with other strains, which underscores the importance of searching for universally protective strains or antigens/antigenic epitopes. Additionally, bacterial challenge routes vary across current studies with some evaluating protection in only a blood or lung infection model. We posit that it will be paramount to evaluate protection in both clinically relevant models.

Novel immunotherapeutics against *A. baumannii* will inform a deeper understanding of its virulence factors and pathogenesis, and elucidating how this microbe evades the host’s innate but not adaptive immune response will further advance therapeutic development. The primary protective correlate of these therapies in current acute infection animal models is antibody-based. Of critical importance, however, is the development of alternative *A. baumannii* challenge models. Current acute models using hypervirulent strains afford little time for the host to mount a robust cell-mediated immune response; models need to be developed to allow *A. baumannii* to successfully infect immunocompetent wild type animals at lower challenge doses without first manipulating the host’s innate immunity. This could come from developing new animal models, or *via* isolating and evaluating new clinical bacterial strains. Extending the time from infection to mortality will facilitate elucidating the cell-mediated aspects of the adaptive immune response and inform the optimal antigen/adjuvant for use in future vaccine formulations.

Recent advancements in vaccine design will provide a novel edge in immunotherapeutic development against this everchanging pathogen. Along these lines, utilization of next-generation genomics, transcriptomics, and proteomics tools will aid identification of conserved virulence factors that can be assessed for their immunoprotective potential. Moreover, *in silico* models will accelerate development of safer protein-based subunit vaccines. We posit that the future will bring safer protein, DNA, and RNA based vaccines constructed to target conserved virulence factors and be multivalent affording broader protection. With antibody-mediated protection seemingly the most effective approach, there will also be an emphasis on monoclonal antibody development for clinical use. The road of translational science from the bench to the clinic is long but is paramount to improve patient outcomes against this multidrug resistant pathogen.

## Author contributions

SJ searched literature and wrote the manuscript. J-JY searched literature and edited the manuscript. JC, C-YH and TF edited the manuscript. BA conceived and edited the manuscript. All authors contributed to the article and approved the submitted version.

## Funding

This study was partially funded by the U.S. National Institutes of Health grants NS117742 to TF and AI135005 to C-YH. This work was also supported by The Jane and Roland Blumberg Endowment fund to BA.

## Acknowledgments

Figures were created with BioRender.com

## Conflict of interest

The authors declare that the research was conducted in the absence of any commercial or financial relationships that could be construed as a potential conflict of interest.

## Publisher’s note

All claims expressed in this article are solely those of the authors and do not necessarily represent those of their affiliated organizations, or those of the publisher, the editors and the reviewers. Any product that may be evaluated in this article, or claim that may be made by its manufacturer, is not guaranteed or endorsed by the publisher.
